# Population-based estimate of sibling risk for preterm birth, preterm premature rupture of membranes, placental abruption and pre-eclampsia

**DOI:** 10.1186/1471-2156-9-44

**Published:** 2008-07-08

**Authors:** Jevon Plunkett, Ingrid Borecki, Thomas Morgan, David Stamilio, Louis J Muglia

**Affiliations:** 1 Department of Pediatrics, Washington University School of Medicine, St. Louis, Missouri 63110, USA; 2 Department of Obstetrics and Gynecology, Washington University School of Medicine, St. Louis, Missouri 63110, USA; 3 Division of Statistical Genomics, Washington University School of Medicine, St. Louis, Missouri 63110, USA; 4 Center for Preterm Birth Research, Washington University School of Medicine, St. Louis, Missouri 63110, USA; 5 Human and Statistical Genetics Program, Washington University School of Medicine, St. Louis, Missouri 63110, USA

## Abstract

**Background:**

Adverse pregnancy outcomes, such as preterm birth, preeclampsia and placental abruption, are common, with acute and long-term complications for both the mother and infant. Etiologies underlying such adverse outcomes are not well understood. As maternal and fetal genetic factors may influence these outcomes, we estimated the magnitude of familial aggregation as one index of possible heritable contributions.

Using the Missouri Department of Health's maternally-linked birth certificate database, we performed a retrospective population-based cohort study of births (1989–1997), designating an individual born from an affected pregnancy as the proband for each outcome studied. We estimated the increased risk to siblings compared to the population risk, using the sibling risk ratio, λ_s_, and sibling-sibling odds ratio (sib-sib OR), for the adverse pregnancy outcomes of preterm birth, preterm premature rupture of membranes (PPROM), placental abruption, and pre-eclampsia.

**Results:**

Risk to siblings of an affected individual was elevated above the population prevalence of a given disorder, as indicated by λ_S _(λ_S _(95% CI): 4.3 (4.0–4.6), 8.2 (6.5–9.9), 4.0 (2.6–5.3), and 4.5 (4.4–4.8), for preterm birth, PPROM, placental abruption, and pre-eclampsia, respectively). Risk to siblings of an affected individual was similarly elevated above that of siblings of unaffected individuals, as indicated by the sib-sib OR (sib-sib OR adjusted for known risk factors (95% CI): 4.2 (3.9–4.5), 9.6 (7.6–12.2), 3.8 (2.6–5.5), 8.1 (7.5–8.8) for preterm birth, PPROM, placental abruption, and pre-eclampsia, respectively).

**Conclusion:**

These results suggest that the adverse pregnancy outcomes of preterm birth, PPROM, placental abruption, and pre-eclampsia aggregate in families, which may be explained in part by genetics.

## Background

In the United States, 12.7% of births occur preterm (< 37 weeks) [1], approximately one-fourth of which occur due to preterm premature rupture of membranes (PPROM) [2]. Pre-eclampsia and placental abruption affect approximately 7% [3] and 1% [4] of all pregnancies, respectively. While many pregnancies share more than one of these complications, together they affect a significant portion of pregnancies and represent the most common reasons for early delivery. Moreover, adverse pregnancy outcomes are important causes of perinatal morbidity and mortality. For example, placental abruption, while uncommon, accounts for 12% of all perinatal deaths [5]. The incidence of preterm birth [1] and placental abruption [5] have increased over recent decades, further motivating additional study to understand susceptibility factors which contribute to these outcomes.

Prediction and prevention of these adverse outcomes is difficult. Etiologies underlying preterm birth, PPROM, placental abruption and pre-eclampsia are not well understood. Genetic studies are one way in which we can attempt to better understand these disorders. Such studies may identify genetic markers that can predict one's risk for a particular pregnancy outcome. Genetic studies may also identify novel proteins and/or pathways involved in the disorder.

Both maternal and fetal genetic factors may influence adverse pregnancy outcomes. Evidence suggests that maternal genetic factors contribute to preterm birth [6,7], PPROM [7-9], placental abruption [10,11] and pre-eclampsia [12-15]. In contrast, fetal effects on these outcomes have not been well studied. Several lines of evidence suggest that fetal genetic effects may influence adverse pregnancy outcomes. First, fetal genes that are paternally imprinted mainly control placental and fetal membrane growth [5]. Because the placenta and fetal membranes likely play a role in adverse pregnancy outcomes, fetal genes controlling these tissues may also contribute. Additionally, heritability studies, which estimate the relative portion of population variation in a trait due to genetics, suggest that preterm birth [16] and pre-eclampsia [17] are influenced in part by fetal genetic factors. Lastly, several studies suggest that paternity affects risk for preterm birth and pre-eclampsia. For example, several studies indicate that partner changes between pregnancies reduce risk of preterm birth [18,19] and pre-eclampsia [17,20-22]. Changes in paternity may reflect association with long interpregnancy intervals rather than paternity effects *per se*; however, for pre-eclampsia [14,23], fathers' family history affects risk for the disorder in their partners' pregnancies. For preterm birth, father's family history has been shown to have only a weak association with risk. While an early study of a Norway birth registry demonstrated a correlation between father and children's gestational ages [24], a more recent and extensive study of this registry suggested fathers contributed little to no risk to preterm delivery risk [25]. Also, paternal race has been associated with preterm birth risk [26,27]. Together, this data suggests that paternal genes expressed in the fetus may contribute to these disorders, motivating study of maternal-fetal influences, assessed by defining the infant as the proband, in addition to influences that are maternal-specific.

While multiple lines of evidence suggest the importance of genetic contributors in adverse pregnancy outcomes, observing clustering of such outcomes in families is necessary to assert genetic influences on a disorder. A disorder that does not aggregate in families is unlikely to be influenced by inherited factors. Hence, detecting an increased risk for a disorder among siblings or other family members of an individual born from a pregnancy affected by the same adverse outcome would further support genetic influences on these conditions. However, familial aggregation is not sufficient to claim genetic influences on a disorder. Since family members share both genes and environment, any similarities seen in families may be explained by genetic or shared environmental factors (such as in utero maternal environment) or by their interaction.

Two standard measures of familial aggregation are increase in risk to siblings of affected individuals, compared to the population risk for the disorder, the sibling risk ratio, λ_s_[28], and compared to siblings of unaffected individuals, the sibling-sibling odds ratio (sib-sib OR) [29]. These measures have been estimated for a variety of disorders, ranging from single locus Mendelian disorders, such as cystic fibrosis [30], to complex disorders, including hypertension [31], type 2 diabetes [32], and myopia [33,34]. These familial aggregation measures have been incompletely documented in pregnancy outcomes. When considering mother as the affected individual, investigators have reported increased risk among first-degree relatives of women affected with preterm birth [35,36], placental abruption [11] and pre-eclampsia [14,37]; however, few of these studies have scaled the increase in risk among relatives by the population prevalence for a given pregnancy outcome (placental abruption [11], pre-eclampsia [37]), as done to calculate λ_S_. Maternal recurrence risk, similar in calculation to the sib-sib OR, has previously been reported for these disorders [9,38-43]. Yet, only one study of preterm birth and PPROM [9] scaled maternal recurrence relative to population prevalence of the disorder and did not consider this measure as an indication of familial aggregation. λ_S_ and sib-sib OR, defining the infant of an affected pregnancy as proband, have not been reported for these disorders. Estimating λ_S_, in which the increased risk for a disorder is scaled by population prevalence, is particularly important, as population prevalence can vary by race. While there may be a significant increase in risk among siblings or a significant maternal recurrence risk, such a risk may reflect high population prevalence, rather than familial effects, *per se*. As a result, calculating λ_S_ may lead to different conclusions that those made by previous reports of maternal recurrence risk. Since individual demographic factors, such as socioeconomic status or body mass index, may also contribute to risk, we calculate sib-sib OR adjusted for important medical and environmental risk factors to assess to what extent genetic effects may account for familial aggregation.

In order to test whether genetic effects may influence these outcomes, our analyses define the infant of an affected pregnancy as the proband. We estimate λ_S_ and sib-sib OR to determine whether each outcome clusters in families.

## Results

### Preterm birth

The population risks for preterm birth at < 35 gestational weeks were estimated as 3.6%, 2.8%, and 7.8%, in all races, whites and blacks, respectively. Among second-born siblings in the sibling subcohort whose older sibling was affected, rates of preterm birth for all races, whites and blacks, respectively, were used to estimate the sibling risk (see Table 1). λ_S _and its 95% CI were 4.3 (4.0–4.6), 4.4 (4.0–4.7), and 2.8 (2.6–3.1) for all races, whites, and blacks, respectively, indicating a significant increase in risk to siblings of preterm birth patients compared to the population.

**Table 1:** λ_S_ and sib-sib OR (with 95% CI) for preterm birth.

	All races	White	Black
Population: preterm birth	9759	6232	3354
Population: N	268103	220728	42899
Population risk	0.036 (0.035–0.037)	0.028 (0.027–0.029)	0.078 (0.075–0.081)
Siblings: sibpairs with both siblings affected	1020	514	489
Siblings: sibpairs with first sibling affected	6522	4181	2210
Sibling risk	0.156 (0.147–0.165)	0.123 (0.113–0.133)	0.221 (0.204–0.238)
λ_S_	4.3 (4.0–4.6)	4.4 (4.0–4.7)	2.8 (2.6–3.1)
Sib-sib unadjusted OR (95% CI)	5.6 (5.2–6.0)	5.7 (5.2–6.3)	3.6 (3.2–4.0)
Sib-sib adjusted OR (95% CI)#	4.2 (3.9–4.5)	5.1 (4.6–5.7)	3.3 (2.9–3.7)

#Adjusted for mother < 20 years old, mother < 12 education, Medicaid (index of low SES), no prenatal care, mother BMI < 20 kg/m^2^, cigarette smoking

Individuals whose older sibling was affected by preterm birth were also at significantly higher risk compared to individuals whose older sibling was unaffected (see Table 1). This increase in risk persisted after adjusting for known risk factors. Adjusted OR with 95% CI were 4.2 (3.9–4.5), 5.1 (4.6–5.7), and 3.3 (2.9–3.7) for all races, whites and blacks, respectively.

### PPROM

The population risks for PPROM were estimated as 0.8%, 0.6% and 1.9%, in all races, whites and blacks, respectively. Among second siblings in the matched sibling sub-cohort whose older sibling was affected, rates of PPROM were used to estimate sibling risk (see Table 2). λ_S _and its 95% confidence interval were 8.19 (6.50–9.88), 6.75 (4.59–8.91), and 6.40 (4.66–8.14) for all races, whites, and blacks, respectively, indicating a significant increase in risk to siblings of PPROM patients compared to the population.

**Table 2:** λ_S_ and sib-sib OR (with 95% CI) for PPROM.

	All Races	White	Black
Population: PPROM	2105	1311	763
Population: N	254740	211308	39190
Population risk	0.008 (0.008–0.008)	0.006 (0.006–0.006)	0.019 (0.018–0.020)
Siblings: sibpairs with both siblings affected	88	37	49
Siblings: sibpairs with first sibling affected	1300	883	393
Sibling risk	0.068 (0.054–0.082)	0.042 (0.029–0.055)	0.125 (0.092–0.158)
λ_S_	8.2 (6.5–9.9)	6.8 (4.6–8.9)	6.4 (4.7–8.1)
Sib-sib unadjusted OR (95% CI)	10.8 (8.6–13.5)	8.8 (6.3–12.4)	8.8 (6.4–12.1)
Sib-sib adjusted OR (95% CI)#	9.6 (7.6–12.2)	8.5 (6.0–12.1)	8.9 (6.4–12.5)

#Adjusted for mother < 20 years old, mother < 12 education, Medicaid (index of low SES), no prenatal care, mother BMI < 20 kg/m^2^, cigarette smoking

Individuals whose older sibling was affected by PPROM were also at significantly higher risk compared to individuals whose older sibling was unaffected (see Table 2). This increase in risk persisted after adjusting for known risk factors. Adjusted OR with 95% CI were 9.6 (7.6–12.2), 8.5 (6.0–12.1), and 8.9 (6.4–12.5) for all races, whites and blacks, respectively.

### Placental abruption

Population rates of placental abruption were estimated as 0.8%, 0.7%, 1.0%, in all races, whites and blacks respectively. Among second siblings in the matched sibling sub-cohort whose older sibling was affected, rates of placental abruption were used to estimate risk to siblings (see Table 3). λ_S _and its 95% confidence interval were 3.95 (2.63–5.27) and 4.93 (3.18–6.68), for all races and whites, respectively, indicating a significant increase in risk to siblings of placental abruption patients compared to the population.

**Table 3:** λ_S_ and sib-sib OR (with 95% CI) for placental abruption.

	All races	White	Black
Population: placental abruption	2050	1579	428
Population: N	268002	220641	42888
Population risk	0.008 (0.008–0.008)	0.007 (0.007–0.007)	0.010 (0.009–0.011)
Siblings: sibpairs with both siblings affected	34	30	4
Siblings: sibpairs with first sibling affected	1124	851	245
Sibling risk	0.030 (0.020–0.040)	0.035 (0.023–0.047)	0.016 (0–0.032)
λ_S_	4.0 (2.6–5.3)	4.9 (3.2–6.7)	1.6 (0.0–3.2)
Sib-sib unadjusted OR (95% CI)	4.1 (2.9–5.8)	5.4 (3.8–7.9)	1.4 (0.5–3.7)
Sib-sib adjusted OR (95% CI)#	3.8 (2.6–5.5)	5.0 (3.4–7.4)	1.2 (0.4–3.9)

#Adjusted for mother < 20 or > 35 years old, mother < 12 education, Medicaid (index of low SES), no prenatal care, mother BMI < 20 kg/m^2^, cigarette smoking, insulin-dependent diabetes mellitus, chronic hypertension, hydraminos/oligohydraminos

We found that individuals whose older sibling was affected by placental abruption were also at significantly higher risk compared to individuals whose older sibling was unaffected (see Table 3). This increase in risk persisted after adjusting for known risk factors. Adjusted OR with 95% CI: 3.8 (2.6–5.5) and 5.0 (3.4–7.4) for all races and whites, respectively. Blacks did not show a significant increase in risk to siblings of placental abruption births either compared to the population (λ_S _= 1.64 (0.04–3.24)) or compared to siblings of births unaffected by this disorder (unadjusted OR: 1.4 (0.5–3.7), adjusted OR: 1.2 (0.4–3.9)).

### Pre-eclampsia

Population rates of pre-eclampsia were estimated as 3.2%, 3.1%, and 4.1%, in all races, whites and blacks, respectively. Among second siblings in the matched sibling sub-cohort whose older sibling was affected, rates of pre-eclampsia were used to calculate sibling risk (see Table 4). λ_S _and its 95% confidence interval were 4.51 (4.24–4.78), 4.52 (4.21–4.83), and 4.11 (3.59–4.63) for all races, whites, and blacks, respectively.

**Table 4:** λ_S_ and sib-sib OR (with 95% CI) for pre-eclampsia.

	All races	White	Black
Population: pre-eclampsia	8600	6749	1736
Population: N	267840	220505	42861
Population risk	0.032 (0.031–0.033)	0.031 (0.031–0.031)	0.041 (0.039–0.043)
Siblings: sibpairs with both siblings affected	1070	821	233
Siblings: sibpairs with first sibling affected	7384	5869	1400
Sibling risk	0.145 (0.137–0.153)	0.140 (0.131–0.149)	0.166 (0.146–0.186)
λ_S_	4.5 (4.2–4.8)	4.5 (4.2–4.8)	4.1 (3.6–4.6)
Sib-sib unadjusted OR (95% CI)	9.2 (8.5–9.9)	10.0 (9.1–10.9)	6.7 (5.7–7.9)
Sib-sib adjusted OR (95% CI)#	8.1 (7.5–8.8)	9.0 (8.2–9.8)	5.8 (4.9–7.0)

#Adjusted for mother < 20 or > 35 years old, mother < 12 education, Medicaid (index of low SES), no prenatal care, mother BMI < 20 kg/m^2^, cigarette smoking, insulin-dependent diabetes mellitus, chronic hypertension

We found that individuals whose older sibling was affected by pre-eclampsia were also at significantly higher risk compared to individuals whose older sibling was unaffected (see Table 4). This increase in risk persisted after adjusting for known risk factors. Adjusted OR with 95% CI were 8.1 (7.5–8.8), 9.0 (8.2–9.8), and 5.8 (4.9–7.0) for all races, whites and blacks, respectively.

## Discussion

We hypothesized that siblings of individuals who were products of pregnancies affected by one of several adverse outcomes, preterm birth, PPROM, placental abruption and pre-eclampsia, would be at increased risk for the same outcome. λ_S_ and sib-sib OR values significantly greater than one indicate that risk to siblings of adverse pregnancy outcome births is elevated compared to the population rate and to the rate in siblings of unaffected individuals, respectively. None of the 95% CI for λ_S_ or sib-sib OR values overlap with one, with the exception of placental abruption in blacks. The lack of evidence for familial aggregation of placental abruption in blacks may be explained by the rarity of the event and the relatively small racial subgroup (see Table 3). These data suggest that genetic and/or environmental risk factors shared among siblings affect these disorders.

Estimates of sib-sib OR are consistent with previous studies of maternal recurrence risk in the Missouri birth certificate database [38,39], and of maternal recurrence risk scaled to the population prevalence for preterm birth [9]. Our estimate of λ_S_ is noticeably smaller than the maternal recurrence risk, scaled by population prevalence of PPROM estimated in [9] (OR (95% CI): 20.6 (4.7, 90.2)). This difference likely reflects the larger and population-based cohort used in our study, in contrast to [9] in which relatively small groups of PPROM (n = 114) and normal term (n = 208) deliveries were selected from a hospital population.

The utility of these measures lies primarily in establishing familial aggregation of a disorder, a prerequisite to claiming genetic influences on any trait. Yet, λ_S_ values may also be used to make tentative assessments of future genetic studies. The magnitude of λ_S_ values may reflect the mode of genetic etiology, influencing future studies' design. For example, for complex disorders, to which multiple genetic and environmental factors likely contribute, reported λ_S_ values range from 1.3–75, with peaks at 3–4 and 10–15 [31]; in contrast, monogenic Mendelian disorders show λ_S_ values an order of magnitude higher or more (eg. cystic fibrosis λ_S_ ~500 [30]). Thus, moderate values for λ_S_, such as those reported for the adverse pregnancy outcomes studied (see tables 1, 2, 3, 4), are consistent with complex genetic and environmental etiologies. Among complex disorders, λ_S_ has been used to estimate the ability of a study to detect specific genes [44]. However, large values of λ_S_ do not necessarily predict linkage [31,45] or association [46] studies' success. Additionally, measures that reflect the strength of a genetic effect detected either by linkage, λ_S_ calculated with respect to a specific locus, or by association, genotype relative risk, γ, which measures the ratio of disease risks between individuals with and those without the susceptibility genotypes, have only an indirect correlation with λ_S_[46]. Moderate λ_S_ values may correspond with high γ values (eg. rheumatoid arthritis [46]) and vice versa. While limitations in interpreting λ_S_ values exist, disorders with similar λ_S_ values to the adverse pregnancy outcomes reported here have had specific genes mapped (eg. hypertension, obesity [31]), suggesting that identification of specific genes influencing these conditions may be possible.

While the increased risk to siblings may be explained in part by shared genetics, some evidence suggests that multiple interacting environment factors can account for familial clustering [47]. Hence, the clustering of multiple non-genetic risk factors in families may account for these results. In order to distinguish genetic from other familial risk factors, we calculated sib-sib OR unadjusted and adjusted for important known environmental risk factors. Overall, the elevated risk to siblings persists after adjustment for such factors. While there may be important non-genetic factors affecting each outcome for which we have not accounted, we believe these results suggest that genetic influences may contribute to each of the adverse pregnancy outcomes studied.

Interestingly, λ_S_ and sib-sib OR estimates in blacks are generally smaller than those for whites. For PROM and pre-eclampsia, the 95% CI for λ_S_ and sib-sib OR estimates for the two racial groups overlap; however, these CI do not overlap for preterm birth or placental abruption. Hence, it is difficult to determine to what extent family clustering of these outcomes may differ among races. Differences in the magnitude of λ_S_ and sib-sib OR estimates between blacks and whites may be explained in part by the higher population prevalence for blacks compared to whites for each outcome studied (non-overlapping 95% CI, see tables 1, 2, 3, 4), which may reflect higher overall rates of genetic and/or environmental risk factors in this population.

The Missouri database provides many of this study's strengths. The large number of first recorded siblings in the population cohort (n = 267,472) and matched sibpairs in the sibling cohort (n = 163,826) provides a large sample size from which to estimate λ_S_ and sib-sib OR. Additionally, because this database represents a population cohort of births, rather than births selected based on any particular pregnancy outcome, biases due to ascertainment and overreporting, which can inflate λ_S_ values [48], should be minimal.

However, using a birth certificate database like this one also presents several limitations. First, complications of labor and delivery and maternal and infant medical conditions recorded in such databases may be underreported [49]; as a result, population and/or sibling risk for a particular disorder may be underestimated, potentially biasing our results. For example, the relative rarity of placental abruption in the population makes concordant sibships, particularly in blacks, rare, thereby reducing sample sizes for risk estimates for this disorder. Additionally, gestational age estimates contained in birth certificate databases are based primarily on the date of the last menstrual period, which may be recalled inaccurately or misclassified due to postconceptional bleeding [49], potentially influencing estimates of preterm birth and PPROM prevalence in this dataset. We also acknowledge that each of the categories of preterm birth that we analyzed may in themselves be rather heterogeneous. For example, initiation of spontaneous labor may result in preterm birth in each of the categories, though for some etiologies, particularly pre-eclampsia, iatrogenic delivery could contribute significantly. Our utilization of a more rigorous definition of preterm birth at less than 35 weeks should minimize the contribution of iatrogenic delivery. A final important limitation to this database is the limited amount of information on race. Maternal race is self-reported and possibly subject to population stratification and/or admixture. Additionally, information on paternal race is incomplete, further affecting the accuracy of infants' reported race.

The Missouri database also does not document relationships between mothers; as a result, similar calculations cannot be made to estimate familial clustering when the mother of an affected pregnancy is considered the proband. Moreover, the database contains little information on fathers, making it impossible to distinguish full from half siblings in most sibships. Because we cannot distinguish siblings that share both maternal and paternal factors from those that share maternal factors only, we cannot assess to what extent the increased risk can be attributed to factors unique to the fetus, rather than those shared with its mother. Due to these limitations, we cannot examine the relative importance of maternal versus fetal genetic effects, studied by Wilcox et al. [25] and Cnattingius et al. [17], for preterm birth and preeclampsia, respectively. Cnattingius et al. [17] reports 20% of variation in preeclampsia risk is due to fetal genetic effects and the combined effect of fetal genetic factors and couple effects are as important as maternal genetic effects. In contrast, Wilcox and colleagues [25] report only a weak association between father's family history and risk for preterm birth (RR (95% CI): 1.12 (1.01–1.25)), which became nonsignificant at earlier gestational ages (RR (95% CI): 1.06 (0.77–1.44). From this trend, the authors conclude that fetal genes may contribute to normal labor, but, not preterm delivery [25]; however, Wilcox and colleagues [25] have relatively few early preterm offspring of early preterm mothers (n = 91) and fathers (n = 39) from which risk was estimated, and do not stratify based upon race/ethnicity. Similarly, a recent study [50] suggested that paternal genetics contributed little to gestational age, but could not refute the possible role of maternally-inherited genes expressed in the fetus. Hence, while paternally-inherited genes may contribute little to preterm birth or other disorders, maternally-inherited genes expressed in the fetus may still be important. Because of our study's limitations, we may be detecting effects due to shared uterine environment, shaped in part by maternal genes, rather than maternally-inherited genes in siblings. Hence, fetal genetic effects may make contributions of lesser magnitude than maternal genetic factors, with fetal genetic factors having a more prominent role in certain etiologies of preterm birth.

## Conclusion

We have observed familial aggregation of preterm birth, PPROM, placental abruption and pre-eclampsia. Overall, siblings are at increased risk for each outcome, even after adjusting for important known environmental risk factors. While the influence of shared unmeasured environmental risk factors on sibling risk cannot, and should not, be discounted, we hypothesize that maternal and/or fetal genetic influences account for some of the increased risk to siblings observed. Moreover, though it is difficult to determine to what extent fetal and maternal effects overlap in these analyses, we postulate that fetal genetic factors may contribute to these disorders and suggest that they are studied further.

## Methods

### Study design

A protocol was approved by the Missouri Department of Health and Senior Services and by Washington University School of Medicine to analyze the state's maternally linked birth-death certificate database. We analyzed this database to assess the recurrence risk for a discrete group of adverse pregnancy outcomes, including preterm birth, preterm premature rupture of membranes (PPROM), placental abruption, and pre-eclampsia, in maternally-linked siblings. Births to the same mother were linked by a unique identifier called a sibship number, described elsewhere [51]. Full siblings and half-siblings resulting from pregnancies in the same mother were not distinguished. All protected health information with personal identifiers was removed before distributing the data for analysis.

This analysis was restricted to births that occurred between 1989 and 1997, since births that occurred before 1989 lacked complete medical and social histories. Fetal deaths occurring at < 20 weeks gestation, multiple gestation pregnancies and individuals with no maternally-linked siblings recorded in the database were excluded from this analysis. After excluding such cases, the remaining cohort consisted of 473,881 births, of which 383,812 (81.2%) were white and 81,889 (17.3%) were black. 267,472 births (220,728 (82.5%) white and 42,899 (16.0%) black) were the first maternally-linked sibling in the database and used to estimate the population prevalence for each outcome.

A second cohort of matched siblings was constructed from this dataset to analyze sibling risk for each outcome. The two oldest siblings born to the same mother during the study period were included. The dataset was not restricted to parity 0 and parity 1 women, in order to be as unbiased as possible in estimating risk for siblings and providing the best index of population prevalence. Additional siblings born to the same mother were excluded to simplify the statistical model. This cohort comprised of 327,652 matched siblings, of which 265,947 (81.2%) were white and 55,555 (17.0%) were black. Second-born siblings whose older sibling was affected by a particular outcome were used to estimate sibling risk for λ_S_ and sib-sib OR.

### Definitions

Preterm birth is defined by the World Health Organization as delivery < 37 weeks [52]. To avoid inclusion of borderline gestational ages which may introduce misclassification bias, we defined preterm birth as delivery < 35 weeks in this study. Information from the last menstrual period and clinical data were used to calculate the best estimate of gestational age. PPROM was defined as births delivered < 35 weeks complicated by premature rupture of membranes. For PPROM, births complicated by pre-eclampsia, insulin-dependent and other diabetes, or eclampsia were excluded from analysis due to the potential for these births being delivered for medical reasons. First-born sibling and second-born sibling refer to the two oldest siblings recorded in database.

### Statistical analysis

$$\lambda s=\frac{\text{P(affected|affected sibling)}}{\text{Population prevalence}}$$

λ_S _was calculated as the frequency of an outcome in the individuals whose older sibling was affected with the disorder in the sibling cohort divided by the frequency of the outcome in first siblings in the larger cohort. 95% confidence intervals (CI) for sibling risk, population risk and sibling risk ratio were calculated by standard procedures for a binomial variable.

$$\text{Sib}-\text{sib OR}=\frac{\text{P(affected|affected sibling)}}{\text{P(affected|unaffected sibling)}}$$

Sib-sib OR was calculated as the odds of a child being affected with a particular adverse outcome, given that their older sibling was affected, divided by the odds of a child being affected with a particular adverse outcome, given that their older sibling was unaffected. Sib-sib OR were adjusted for known medical and environmental risk factors for the outcome to most conservatively estimate residual familial effects on risk. For preterm-birth and PPROM, OR were adjusted for: mother's age < 20 years old, mother < 12 years of education, recipient of state-funded assistance (an index of low socioeconomic status), no prenatal care, mother's body mass index (BMI) < 20 kg/m^2^, and cigarette smoking during pregnancy. In addition to these risk factors, pre-eclampsia ORs were corrected for: mother's age > 35 years old, insulin-dependent diabetes mellitus, chronic hypertension. ORs for placental abruption were corrected for hydraminos/oligohydraminos in addition to the risk factors listed above.

Frequencies for λ_S _and logistic regression analyses for the sib-sib OR were performed using Stata 9 [53]. Each calculation was made for preterm birth, PPROM, placental abruption, and pre-eclampsia in all races (including individuals whose race was neither black nor white), as well as stratified by black or white race. λ_S _and sib-sib ORs calculated by race compare siblings of affected individuals designated as black or white to the siblings of unaffected individuals of the same race or the population prevalence for that race.

## Authors' contributions

JP participated in the design of the study, carried out the statistical analyses, participated in interpreting results, and drafted the manuscript. IB participated in interpreting results and contributed to the manuscript. TM and DS participated in the design of the study, provided support for the statistical analysis, participated in interpreting results, and contributed to the manuscript. LJM conceived of the study, participated in its design and coordination, and contributed to the manuscript. All authors read and approved the final manuscript.

## References

[B1] Hamilton BE, Minino AM, Martin JA, Kochanek KD, Strobino DM, Guyer B (2007). Annual summary of vital statistics: 2005. Pediatrics.

[B2] Tucker JM, Goldenberg RL, Davis RO, Copper RL, Winkler CL, Hauth JC (1991). Etiologies of preterm birth in an indigent population: is prevention a logical expectation?. Obstet Gynecol.

[B3] Carr DB, Epplein M, Johnson CO, Easterling TR, Critchlow CW (2005). A sister's risk: family history as a predictor of preeclampsia. American journal of obstetrics and gynecology.

[B4] Hladky K, Yankowitz J, Hansen WF (2002). Placental abruption. Obstetrical & gynecological survey.

[B5] Ananth CV, Wilcox AJ (2001). Placental abruption and perinatal mortality in the United States. American journal of epidemiology.

[B6] Crider KS, Whitehead N, Buus RM (2005). Genetic variation associated with preterm birth: a HuGE review. Genet Med.

[B7] DeFranco E, Teramo K, Muglia L (2007). Genetic influences on preterm birth. Seminars in reproductive medicine.

[B8] Doody DR, Patterson MQ, Voigt LF, Mueller BA (1997). Risk factors for the recurrence of premature rupture of the membranes. Paediatric and perinatal epidemiology.

[B9] Lee T, Carpenter MW, Heber WW, Silver HM (2003). Preterm premature rupture of membranes: risks of recurrent complications in the next pregnancy among a population-based sample of gravid women. American journal of obstetrics and gynecology.

[B10] Ananth CV, Savitz DA, Williams MA (1996). Placental abruption and its association with hypertension and prolonged rupture of membranes: a methodologic review and meta-analysis. Obstetrics and gynecology.

[B11] Toivonen S, Keski-Nisula L, Saarikoski S, Heinonen S (2004). Risk of placental abruption in first-degree relatives of index patients. Clinical genetics.

[B12] Nilsson E, Salonen Ros H, Cnattingius S, Lichtenstein P (2004). The importance of genetic and environmental effects for pre-eclampsia and gestational hypertension: a family study. BJOG.

[B13] Salonen Ros H, Lichtenstein P, Lipworth L, Cnattingius S (2000). Genetic effects on the liability of developing pre-eclampsia and gestational hypertension. American journal of medical genetics.

[B14] Skjaerven R, Vatten LJ, Wilcox AJ, Ronning T, Irgens LM, Lie RT (2005). Recurrence of pre-eclampsia across generations: exploring fetal and maternal genetic components in a population based cohort. BMJ (Clinical research ed.

[B15] Thornton JG, Macdonald AM (1999). Twin mothers, pregnancy hypertension and pre-eclampsia. British journal of obstetrics and gynaecology.

[B16] Lunde A, Melve KK, Gjessing HK, Skjaerven R, Irgens LM (2007). Genetic and environmental influences on birth weight, birth length, head circumference, and gestational age by use of population-based parent-offspring data. American journal of epidemiology.

[B17] Cnattingius S, Reilly M, Pawitan Y, Lichtenstein P (2004). Maternal and fetal genetic factors account for most of familial aggregation of preeclampsia: a population-based Swedish cohort study. Am J Med Genet A.

[B18] Li DK (1999). Changing paternity and the risk of preterm delivery in the subsequent pregnancy. Epidemiology (Cambridge, Mass.

[B19] Vatten LJ, Skjaerven R (2003). Effects on pregnancy outcome of changing partner between first two births: prospective population study. BMJ (Clinical research ed.

[B20] Mostello D, Kallogjeri D, Tungsiripat R, Leet T (2008). Recurrence of preeclampsia: effects of gestational age at delivery of the first pregnancy, body mass index, paternity, and interval between births. Am J Obstet Gynecol.

[B21] Trogstad LI, Eskild A, Magnus P, Samuelsen SO, Nesheim BI (2001). Changing paternity and time since last pregnancy; the impact on pre-eclampsia risk. A study of 547 238 women with and without previous pre-eclampsia. International journal of epidemiology.

[B22] Zhang J, Patel G (2007). Partner change and perinatal outcomes: a systematic review. Paediatric and perinatal epidemiology.

[B23] Esplin MS, Fausett MB, Fraser A, Kerber R, Mineau G, Carrillo J, Varner MW (2001). Paternal and maternal components of the predisposition to preeclampsia. The New England journal of medicine.

[B24] Lie RT, Wilcox AJ, Skjaerven R (2006). Maternal and paternal influences on length of pregnancy. Obstet Gynecol.

[B25] Wilcox AJ, Skaerven R, Lie RT (2008). Familial patterns of preterm delivery: maternal and fetal contributions. American journal of epidemiology.

[B26] Palomar L, DeFranco EA, Lee KA, Allsworth JE, Muglia LJ (2007). Paternal race is a risk factor for preterm birth. American journal of obstetrics and gynecology.

[B27] Tan H, Wen SW, Walker M, Demissie K (2004). Parental race, birth weight, gestational age, and fetal growth among twin infants in the United States. Early human development.

[B28] Risch N (1990). Linkage strategies for genetically complex traits. I. Multilocus models. American journal of human genetics.

[B29] La Batide-Alanore A, Tregouet DA, Jaquet D, Bouyer J, Tiret L (2002). Familial aggregation of fetal growth restriction in a French cohort of 7,822 term births between 1971 and 1985. American journal of epidemiology.

[B30] Lander ES, Schork NJ (1994). Genetic dissection of complex traits. Science (New York, NY.

[B31] Altmuller J, Palmer LJ, Fischer G, Scherb H, Wjst M (2001). Genomewide scans of complex human diseases: true linkage is hard to find. American journal of human genetics.

[B32] Weijnen CF, Rich SS, Meigs JB, Krolewski AS, Warram JH (2002). Risk of diabetes in siblings of index cases with Type 2 diabetes: implications for genetic studies. Diabet Med.

[B33] Farbrother JE, Kirov G, Owen MJ, Guggenheim JA (2004). Family aggregation of high myopia: estimation of the sibling recurrence risk ratio. Investigative ophthalmology & visual science.

[B34] Fotouhi A, Etemadi A, Hashemi H, Zeraati H, Bailey-Wilson JE, Mohammad K (2007). Familial aggregation of myopia in the Tehran eye study: estimation of the sibling and parent offspring recurrence risk ratios. The British journal of ophthalmology.

[B35] Porter TF, Fraser AM, Hunter CY, Ward RH, Varner MW (1997). The risk of preterm birth across generations. Obstetrics and gynecology.

[B36] Winkvist A, Mogren I, Hogberg U (1998). Familial patterns in birth characteristics: impact on individual and population risks. International journal of epidemiology.

[B37] Dawson LM, Parfrey PS, Hefferton D, Dicks EL, Cooper MJ, Young D, Marsden PA (2002). Familial risk of preeclampsia in Newfoundland: a population-based study. J Am Soc Nephrol.

[B38] Ananth CV, Getahun D, Peltier MR, Salihu HM, Vintzileos AM (2006). Recurrence of spontaneous versus medically indicated preterm birth. American journal of obstetrics and gynecology.

[B39] Ananth CV, Peltier MR, Chavez MR, Kirby RS, Getahun D, Vintzileos AM (2007). Recurrence of ischemic placental disease. Obstet Gynecol.

[B40] Kilpatrick SJ, Patil R, Connell J, Nichols J, Studee L (2006). Risk factors for previable premature rupture of membranes or advanced cervical dilation: a case control study. American journal of obstetrics and gynecology.

[B41] Ladfors L, Mattsson LA, Eriksson M, Milsom I (2000). Prevalence and risk factors for prelabor rupture of the membranes (PROM) at or near-term in an urban Swedish population. Journal of perinatal medicine.

[B42] Lindqvist PG, Happach C (2006). Risk and risk estimation of placental abruption. European journal of obstetrics, gynecology, and reproductive biology.

[B43] Rasmussen S, Irgens LM, Dalaker K (1997). The effect on the likelihood of further pregnancy of placental abruption and the rate of its recurrence. Br J Obstet Gynaecol.

[B44] Risch N (1990). Linkage strategies for genetically complex traits. II. The power of affected relative pairs. American journal of human genetics.

[B45] Guo SW (2002). Sibling recurrence risk ratio as a measure of genetic effect: caveat emptor!. American journal of human genetics.

[B46] Rybicki BA, Elston RC (2000). The relationship between the sibling recurrence-risk ratio and genotype relative risk. American journal of human genetics.

[B47] Guo SW (2000). Familial aggregation of environmental risk factors and familial aggregation of disease. American journal of epidemiology.

[B48] Guo SW (1998). Inflation of sibling recurrence-risk ratio, due to ascertainment bias and/or overreporting. American journal of human genetics.

[B49] Schoendorf KC, Branum AM (2006). The use of United States vital statistics in perinatal and obstetric research. American journal of obstetrics and gynecology.

[B50] Kistka ZA, Defranco EA, Ligthart L, Willemsen G, Plunkett J, Muglia LJ, Boomsma DI (2008). Heritability of parturition timing: an extended twin design analysis. Am J Obstet Gynecol.

[B51] Herman AA, McCarthy BJ, Bakewell JM, Ward RH, Mueller BA, Maconochie NE, Read AW, Zadka P, Skjaerven R (1997). Data linkage methods used in maternally-linked birth and infant death surveillance data sets from the United States (Georgia, Missouri, Utah and Washington State), Israel, Norway, Scotland and Western Australia. Paediatric and perinatal epidemiology.

[B52] World Health Organization WHO (1992). International statistical classification of diseases and related health problems.

[B53] StataCorp (2005). Stata Statistical Software: Release 9..

